# Trend and age-period-cohort analysis of colorectal cancer burden attributable to low whole grains intake: a global, regional, and national analysis with predictive modeling (1990–2046)

**DOI:** 10.3389/fnut.2025.1632103

**Published:** 2025-12-12

**Authors:** Kai Wang, Xiaodan Li, Zhiqiang Guo, Junsheng Chen, Yongsheng Li, Hongzhou Liu, Shuwei Guo

**Affiliations:** 1 Department of Colorectal Surgery, Heping Hospital Affiliated to Changzhi Medical College, Changzhi, Shanxi, China; 2 Department of Pediatric Health Care, Zhangzi County Maternal and Child Health Family Planning Service Center, Changzhi, Shanxi, China

**Keywords:** colorectal cancer (CRC), global burden of disease, low whole grains intake, death, disability-adjusted life years (DALYs), age-period-cohort (APC) model

## Abstract

**Background:**

Colorectal cancer (CRC) persists as a major global public health priority, with insufficient whole grain consumption recognized as a clinically significant and modifiable dietary risk factor. However, comprehensive analyses of its spatiotemporal burden and socioeconomic disparities are limited. This study evaluates the global, regional, and national CRC burden attributable to low whole grains intake from 1990 to 2021 and projects trends to 2046.

**Methods:**

This study employed the Global Burden of Disease (GBD) 2021 dataset to quantify the association between low whole grain intake and CRC burden. Analyses were conducted across multiple dimensions: temporal (1990–2021), demographic (stratified by sex and 5-year age intervals), and geographic (categorized by national and regional Socio-demographic Index (SDI) quintiles).

**Results:**

From 1990 to 2021, global CRC deaths and disability-adjusted life years (DALYs) attributable to low whole grains intake increased by 82.94% (101,813 to 186,257) and 70.30% (2,540,867 to 4,327,219), respectively. However, age-standardized mortality rates (ASMR) and age-standardized DALYs rates (ASDR) declined globally (AAPC: −0.73 and −0.74). High-SDI regions showed the steepest reductions (ASMR AAPC: −1.17; ASDR AAPC: −1.15), while low-middle-SDI regions experienced rising trends (ASMR AAPC: 0.42; ASDR AAPC: 0.35). Gender disparities persisted: males had higher absolute burdens (2021 deaths: 104,344 vs. 81,912 for females; 2021DALYs: 2527992 vs. 1,799,227), but female ASMR and ASDR declined faster (AAPC: −1.09, −1.12, respectively, vs. −0.47 and −0.47, respectively, for males). Geographically, East Asia, Western Europe, and High-income North America had the highest absolute burdens, whereas Uruguay (ASMR: 5.18/100,000) and Hungary (ASDR: 113.76/100,000) led in age-standardized rates. Cabo Verde exhibited the sharpest increases (ASMR AAPC: 3.16; ASDR AAPC: 2.80). Frontier analysis identified Uruguay and Hungary as high-SDI countries with the largest gaps from efficiency targets. Projections to 2046 suggest continued ASMR/ASDR declines but persistent SDI-driven disparities.

**Conclusion:**

Despite declining age-standardized rates, absolute CRC burden attributable to low whole grains intake increased due to population growth and aging. Socioeconomic disparities highlight the need for targeted interventions in transitioning regions adopting Western diets. Promoting whole grains consumption and prioritizing high-burden populations could mitigate future CRC burden.

## 1 Introduction

Colorectal cancer (CRC) is one of the most common malignancies worldwide, with incidence and mortality rates steadily increasing in recent years (1–3) According to GLOBOCAN 2022 data, CRC ranks third among newly diagnosed cancers globally and second in terms of cancer-related deaths, posing significant challenges to global public health (4). The epidemiological characteristics of CRC vary markedly across regions, age groups, genders, and racial populations, partly attributable to differences in exposure to risk factors, demographic shifts, and genetic susceptibility (5–7). With economic growth and Westernization of lifestyles, the incidence of CRC is rapidly increasing in many low- and middle-income countries, particularly in Eastern Europe, Asia, and South America (8–10).

To date, dietary factors have been recognized as important determinants of CRC risk, with growing evidence that low whole-grain intake is associated with higher CRC incidence (11). Whole grains are rich in dietary fiber, vitamins, and minerals, which can improve gut health and reduce inflammation, potentially decreasing the risk of CRC (3, 12). Despite these mechanistic insights, global data show that low whole-grain consumption remains prevalent and is contributing to CRC burden across regions (13).

Although existing studies indicate that low whole grains intake is significantly correlated with elevated CRC incidence, epidemiological research on this dietary pattern on a global scale remains limited. Particularly in low- and middle-income countries, the widespread adoption of Western dietary patterns may exacerbate the burden of CRC as low whole grains intake becomes more common (14–16). Given regional and population differences in dietary patterns, a systematic evaluation of the CRC burden attributable to low whole-grain intake is of substantial public health importance, informing dietary guidelines and targeted prevention efforts.

The Global Burden of Disease (GBD) study covers 371 diseases and 88 related risk factors, providing data support for exploring and elucidating the epidemiological characteristics of CRC (17–19). We obtained detailed figures, percentages, and ratios of CRC deaths and disability-adjusted life years (DALYs) attributable to low whole grains diets from the GBD 2021 database. Our analysis focuses on examining temporal trends in CRC burden and elucidating how gender, age, geographic location, and the Sociodemographic Index (SDI) influence deaths and DALY attributable to low whole grains intake. Additionally, we conducted projections to anticipate future trends, providing valuable insights to strengthen CRC prevention strategies.

## 2 Methods

### 2.1 Data sources and GBD framework overview

The GBD 2021 study estimated data for 371 diseases and 88 related risk factors at the global, regional, and national levels (19). In this study, we utilized the GBD 2021 database to identify the most significant dietary risk factor for CRC: “Low whole grains” Subsequently, we selected “Colorectal cancer” from the “Tumors” list. Data on CRC deaths, DALY, age-standardized death rates (ASMR), and age-standardized DALY rates (ASDR) attributable to low whole grains intake were extracted from the GBD Results Tool^1^. These indicators were analyzed across 21 GBD regions and 204 countries from 1990 to 2021, including information on gender, age, and the SDI (19).

All estimates derived from the GBD study, including death counts, DALYs, and age-standardized rates, are presented with their corresponding 95% uncertainty intervals (UIs). Based on the GBD framework, 95% UI for all estimates were calculated by averaging the data from 1,000 draws, with the lower and upper bounds of the 95% UIs established by the 25th and 975th ranked values among all 1,000 draws (20, 21).

The GBD hierarchy organizes locations into three levels: super regions, regions, and countries. They divided countries into 21 regions on the basis of two criteria: epidemiological homogeneity, and geographical contiguity. For some statistical analyses, they grouped regions into 7 super-regions: High-income, Central Europe Eastern Europe and Central Asia, Latin America and Caribbean, Southeast Asia East Asia and Oceania, South Asia, North Africa and Middle East, and Sub-Saharan Africa (22).

The SDI is a composite indicator reflecting a region or country’s per capita income, fertility rate, and educational attainment. Based on SDI values (ranging from 0 to 1), regions and countries were classified into five groups: high SDI (>0.81), high-middle SDI (0.70–0.81), middle SDI (0.61–0.69), low-middle SDI (0.46–0.60), and low SDI (<0.46) (23–25).

### 2.2 Definition of disease and risk factors

The CRC was identified using the following international classification of diseases, tenth edition (ICD10) codes: C18C19.0, C20, C21-C21.8, Z12.1-Z12.13, Z85.03-Z85.048, Z86.010 (19).

Whole grains intake refers to the average daily consumption, in grams, of whole grains and their products, including breakfast cereals, bread, rice, cookies, muffins, corn cakes, pancakes, pasta, and other related foods. Diets with daily whole grains intake below 140–160 grams are considered to be low in whole grains (26, 27).

### 2.3 Statistical analyses

We conducted a descriptive analysis to illustrate the overall burden of CRC attributable to low whole grains intake. The analysis included the number of deaths, DALY, ASMR, and ASDR. This study characterized the burden of CRC associated with low whole grains intake across different SDI levels, geographic regions, countries, genders, and age groups by analyzing death counts and DALY. Trends over time were assessed using ASMR and ASDR.

The Joinpoint software (version 5.3.0.0; National Cancer Institute) was used to calculate the Average Annual Percentage Change (AAPC) to evaluate trends in disease burden. A regression model was applied to the natural logarithm of age-standardized indicators, using the formula ln(y) = *α* + *β* x + *ε*, where *y* represents the age-standardized metric and x is the calendar year. The AAPC was calculated as 100 × (exp(*β*) − 1). The 95% confidence intervals (CIs) for the AAPC were derived from the standard error of the estimated regression coefficient β (in multi-segment models, the SE is obtained by the appropriate combining rule) and were computed by the software under an asymptotic normal approximation. (28). An AAPC with a 95% CI above zero indicates an increasing trend; below zero indicates a decreasing trend; and if the interval includes zero, the trend is considered stable. It is important to note that the Joinpoint regression analysis was performed on the age-standardized rates (ASMR and ASDR) point estimates obtained from GBD. The 95% CIs for the AAPC reflect the uncertainty in the trend estimation itself, and are distinct from the 95% UIs provided by GBD for each annual rate estimate, which reflect the uncertainty in the data and modeling of the rates.

Frontier analysis evaluates the performance and efficiency of decision-making units (such as countries or regions) in converting inputs into outputs (29). We used Data Envelopment Analysis (DEA) and Stochastic Frontier Analysis (SFA) to construct efficiency frontiers representing best practices, comparing each decision unit’s performance to these frontiers to determine efficiency levels. The performance of countries and regions with varying SDI levels was assessed, with the lower bounds of ASMR and ASDR representing the minimum achievable values given each SDI level.

The age–period–cohort (APC) model was used to evaluate the independent effects of age, period, and cohort on mortality rates and DALYs rates. The analysis was conducted in five-year intervals, with the indicators including the relative risk (RR) for period and cohort effects. Since the CRC mortality rate and DALY rate attributed to low whole grains intake were zero among populations under 25 years old, this group was excluded from the model analysis in this study.

The Bayesian Age-Period-Cohort (BAPC) model is a Bayesian statistical approach that analyzes and forecasts demographic data by simulating the individual effects of age, period, and birth cohort (30). In the BAPC model, the age effect reflects changes in the risk of events (such as disease onset) as individuals age. Period effects account for external factors such as environmental or policy changes that influence all individuals at specific time points. Cohort effects refer to characteristics inherent to individuals born in a particular period, due to their unique experiences and exposures. We used the BAPC R package to fit the model and project global age-standardized rates (ASRs) by gender up to 2046 (31).

Spearman’s rank correlation test was used to examine the relationship between CRC burden attributable to low whole grains intake (ASMR and ASDR) in 2021 and SDI. Statistical analyses and data visualization were performed using R (version 4.4.1) and Joinpoint (version 5.3.0.0). A *p*-value < 0.05 was considered statistically significant. Our data were obtained from the GBD 2021 Results website (see Footnote 1), which provides publicly accessible, free data. Since ethical approval was obtained for the original study, our research did not require additional ethical approval.

## 3 Results

### 3.1 Global CRC burden attributable to low whole grains intake

Between 1990 and 2021, global CRC deaths linked to low whole grains intake exhibited a substantial increase, rising from 101,813 (95% UI: 42,588 to 151,170) to 186,257 (95% UI: 76,127 to 284,803), representing an increase of 82.94%. Meanwhile, DALYs associated with this dietary risk factor climbed from 2,540,867 (95% UI: 1,050,794 to 3,754,416) to 4,327,219 (95% UI: 1,754,865 to 6,578,232), a rise of 70.30%. During the same period, both ASMR and ASDR showed declines, with AAPCs of −0.73 (95% CI: −0.83 to −0.63) and −0.74 (95% CI: −0.85 to −0.64), respectively. Over the same period, the CRC ASMR and ASDR linked to this dietary risk factor decreased in both males and females globally, with more pronounced declines observed in females; the AAPCs were −1.09 (95% CI: −1.19 to −0.99) and −1.12 (95% CI: −1.21 to −1.03), respectively.

At the SDI regional level, ASMR and ASDR were consistently highest in high and high-middle SDI regions in both 1990 and 2021. From 1990 to 2021, high, high-middle, middle, and low SDI regions showed decreasing trends in CRC ASMR and ASDR attributable to low whole grains intake. The most significant decline was in high SDI region, with AAPCs of −1.17 (95% CI: −1.31 to −1.04) for ASMR and −1.15 (95% CI: −1.28 to −1.01) for ASDR. The middle SDI region experienced the slowest declines, with an ASMR AAPC of −0.08 (95% CI: −0.24 to 0.09) and an ASDR AAPC of −0.19 (95% CI: −0.31 to −0.08). Conversely, the low-middle SDI region showed increasing trends, with an ASMR AAPC of 0.42 (95% CI: 0.33 to 0.51) and an ASDR AAPC of 0.35 (95% CI: 0.29 to 0.40).

From 1990 to 2021, CRC deaths and DALYs attributable to low whole grains intake showed significant increases across 21 GBD regions. The top three regions with the highest CRC deaths and DALYs due to low whole grains intake were East Asia, Western Europe, and High-income North America, while Oceania had the lowest values. Central Sub-Saharan Africa, Southern Sub-Saharan Africa, and Andean Latin America also had notable lower values. The most pronounced increases in ASMR were observed in Southern Sub-Saharan Africa, Central Latin America, and Southeast Asia, with AAPCs ranging from 0.69 to 0.92. Similarly, the top three regions for ASDR increases were Central Latin America, Southern Sub-Saharan Africa, and Tropical Latin America, with AAPCs between 0.73 and 1.11. In contrast, Australasia experienced the largest declines in ASMR and ASDR, with AAPCs of −1.64 (95% CI: −1.83 to −1.44) and −1.76 (95% CI: −1.93 to −1.58), respectively (Table 1).

**Table 1:** Global burden of CRC attributable to low whole grains intake in 1990 and 2021, with Average Annual Percentage Change (AAPC).

Characteristics	1990				2021				AAPC (1990–2021)	
	Deaths cases	ASMR per 100,000	DALYs	ASDR per 100,000	Deaths cases	ASMR per 100,000	DALYs	ASDR per 100,000	ASMR	ASDR
	No. (95% UI)	No. (95% UI)	No. (95% UI)	No. (95% UI)	No. (95% UI)	No. (95% UI)	No. (95% UI)	No. (95% UI)	No. (95% CI)	No. (95% CI)
Global	101,813 (42,588, 151,170)	2.79 (1.17, 4.15)	2,540,867 (1,050,794, 3,754,416)	63.47 (26.35, 93.84)	186,257 (76,127, 284,803)	2.21 (0.91, 3.38)	4,327,219 (1,754,865, 6,578,232)	50.19 (20.37, 76.30)	−0.73 (−0.83, −0.63)	−0.74 (−0.85, −0.64)
Sex										
Male	51,622 (21,646, 76,728)	3.19 (1.35, 4.78)	1,349,763 (563,930, 2,011,923)	72.49 (30.36, 107.77)	104,344 (42,110, 159,294)	2.75 (1.11, 4.20)	2,527,992 (1,020,944, 3,876,411)	62.39 (25.20, 95.64)	−0.47 (−0.59, −0.34)	−0.47 (−0.57, −0.38)
Female	50,191 (20,942, 74,790)	2.47 (1.04, 3.69)	1,191,104 (486,865, 1,765,199)	55.85 (22.91, 82.83)	81,912 (34,184, 123,364)	1.76 (0.74, 2.66)	1,799,227 (736,335, 2,717,497)	39.36 (16.10, 59.45)	−1.09 (−1.19, −0.99)	−1.12 (−1.21, −1.03)
SDI region										
High SDI	43,043 (18,345, 64,229)	3.87 (1.65, 5.77)	941,428 (398,133, 1,402,354)	86.30 (36.51, 128.53)	60,473 (25,200, 92,184)	2.70 (1.12, 4.10)	1,205,653 (500,387, 1,815,640)	60.69 (25.12, 91.16)	−1.17 (−1.31, −1.04)	−1.15 (−1.28, −1.01)
High-middle SDI	31,690 (13,146, 46,873)	3.36 (1.40, 4.98)	808,498 (332,629, 1,194,207)	80.20 (33.06, 118.50)	56,721 (22,911, 86,095)	2.89 (1.17, 4.38)	1,298,820 (519,592, 1,965,736)	66.60 (26.65, 100.77)	−0.47 (−0.68, −0.26)	−0.57 (−0.78, −0.36)
Middle SDI	18,435 (7,398, 28,070)	1.89 (0.77, 2.87)	537,904 (215,134, 819,573)	47.61 (19.10, 72.49)	47,738 (19,197, 73,112)	1.85 (0.74, 2.83)	1,230,182 (494,733, 1,879,459)	44.72 (17.98, 68.32)	−0.08 (−0.24, 0.09)	−0.19 (−0.31, −0.08)
Low-middle SDI	5,748 (2,417, 8,914)	0.98 (0.41, 1.52)	170,154 (70,941, 262,876)	25.28 (10.62, 39.13)	15,442 (6,372, 22,974)	1.11 (0.46,1.65)	430,073 (176,445, 643,410)	28.05 (11.53, 41.95)	0.42 (0.33, 0.51)	0.35 (0.29, 0.40)
Low SDI	2,751 (1,161, 4,291)	1.29 (0.54, 2.02)	79,389 (33,441, 123,673)	32.35 (13.68, 50.39)	5,640 (2,327, 8,440)	1.23 (0.51, 1.84)	157,203 (64,962, 233,832)	28.75 (11.87, 42.88)	−0.14 (−0.28, −0.01)	−0.38 (−0.47, −0.29)
GBD regions										
Andean Latin America	311 (133, 475)	1.61 (0.69, 2.46)	7,809 (3,322, 11,900)	36.67 (15.64, 55.90)	1,024 (392, 1,605)	1.77 (0.68, 2.78)	23,979 (9,183, 37,638)	40 (15.30, 62.81)	0.33 (−0.22, 0.88)	0.30 (−0.25, 0.86)
Australasia	1,009 (421, 1,533)	4.35 (1.82, 6.61)	23,317 (9,745, 35,119)	100.80 (42.14, 151.66)	1,479 (602, 2,259)	2.61 (1.08, 4.00)	29,863 (12,591, 45,527)	58.54 (25.00, 88.69)	−1.64 (−1.83, −1.44)	−1.76 (−1.93, −1.58)
Caribbean	637 (272, 960)	2.57 (1.09, 3.86)	15,190 (6,477, 22,950)	57.97 (24.72, 87.64)	1,426 (562, 2,167)	2.64 (1.04, 4.00)	32,389 (12,894, 49,039)	60.31 (24.05, 91.36)	0.21 (0.17, 0.25)	0.18 (−0.01, 0.37)
Central Asia	908 (379, 1,365)	1.95 (0.82, 2.94)	25,987 (10,919, 39,252)	52.45 (22.00, 79.19)	1,163 (481, 1784)	1.49 (0.62, 2.29)	31,919 (13,174, 48,964)	36.92 (15.25, 56.58)	−0.73 (−1.20, −0.27)	−0.97 (−1.10, −0.84)
Central Europe	5,915 (2,478, 8,741)	4.06 (1.70, 6.00)	141,072 (58,975, 208,942)	94.12 (39.32,139.42)	9,495 (3,942, 14,240)	4.13 (1.71, 6.20)	199,028 (82,275, 297,982)	92.49 (38.15, 138.76)	0.06 (−0.03, 0.14)	−0.05 (−0.14, 0.03)
Central Latin America	926 (380, 1,366)	1.20 (0.49, 1.77)	24,187 (9,946,35, 390)	27.34 (11.24, 40.18)	3,795 (1,513, 5,783)	1.54 (0.61, 2.34)	97,676 (39,089, 148,606)	38.15 (15.26, 58.09)	0.86 (0.62, 1.09)	1.11 (1.03, 1.18)
Central Sub-Saharan Africa	256 (109, 397)	1.28 (0.55, 1.99)	7,454 (3,138, 11,527)	30.78 (13.03, 47.79)	645 (261, 1,088)	1.31 (0.54, 2.23)	18,961 (7,606, 31,747)	31.16 (12.64, 52.56)	0.09 (0.03, 0.15)	0.04 (−0.03, 0.11)
East Asia	22,088 (8,771, 34,016)	2.78 (1.10, 4.27)	646,779 (257,106, 994,560)	69.28 (27.54, 106.65)	52,283 (20,976, 83,298)	2.50 (1.00, 3.97)	1,295,744 (524,660, 2,051,943)	60.18 (24.33, 95.13)	−0.34 (−0.49, −0.19)	−0.45 (−0.57, −0.33)
Eastern Europe	9,453 (3,920, 14,006)	3.41 (1.41, 5.04)	240,975 (99,959, 357,627)	85.55 (35.48, 126.94)	12,007 (5,039, 17,766)	3.36 (1.41, 4.98)	273,010 (114,999, 401,921)	78.90 (33.27, 116.43)	−0.02 (−0.46, 0.42)	−0.22 (−0.71, 0.27)
Eastern Sub-Saharan Africa	1,365 (575, 2,131)	1.95 (0.82, 3.04)	38,964 (16,326, 60,927)	48.22 (20.27, 75.34)	2,774 (1,189, 4,140)	1.89 (0.82, 2.82)	75,703 (31,963, 112,169)	42.13 (18.01, 62.87)	−0.11 (−0.23, 0.01)	−0.44 (−0.54, −0.34)
High-income Asia Pacific	5,843 (2,461, 8,831)	3.03 (1.28, 4.59)	142,754 (59,701, 214,811)	70.50 (29.53, 106.10)	13,974 (5,931, 21,445)	2.60 (1.09, 3.96)	250,021 (105,027, 380,218)	57.34 (23.88, 86.30)	−0.51 (−0.78, −0.24)	−0.68 (−0.89, −0.47)
High-income North America	13,194 (5,633, 19,617)	3.67 (1.57, 5.45)	286,863 (121,592, 423,918)	83.39 (35.33, 123.23)	15,390 (6,564, 23,033)	2.32 (0.99, 3.46)	342,013 (144,494, 508,975)	56.56 (23.83, 84.12)	−1.51 (−1.61, −1.40)	−1.26 (−1.51, −1.02)
North Africa and Middle East	2,638 (1,070, 3,947)	1.69 (0.69, 2.53)	75,303 (31,144, 112,922)	41.27 (16.87, 62.03)	7,010 (2,867, 10,717)	1.68 (0.69, 2.57)	188,025 (75,808, 285,507)	38.97 (15.81, 59.47)	−0.01 (−0.15, 0.13)	−0.19 (−0.30, −0.07)
Oceania	30 (12, 48)	1.17 (0.47, 1.81)	933 (373, 1,455)	28.41 (11.49, 44.39)	70 (28, 107)	1.03 (0.41, 1.56)	2,114 (840, 3,231)	25.10 (9.99, 38.13)	−0.41 (−0.52, −0.31)	−0.42 (−0.58, −0.26)
South Asia	4,280 (1,859, 6,564)	0.76 (0.33, 1.17)	131,068 (56,845, 200,092)	20.12 (8.75, 30.74)	11,356 (4,736, 17,077)	0.79 (0.33, 1.18)	320,918 (133,327, 481,047)	20.32 (8.45, 30.54)	0.16 (−0.01, 0.32)	0.05 (−0.05, 0.15)
Southeast Asia	3,950 (1,640, 6,043)	1.62 (0.68, 2.49)	115,594 (47,680, 177,860)	41.08 (17.03, 63.08)	12,528 (5,042, 18,993)	2.01 (0.81, 3.04)	340,389 (136,102, 511,101)	49.19 (19.70, 74.27)	0.69 (0.55, 0.83)	0.57 (0.54, 0.61)
Southern Latin America	1,697 (710, 2,525)	3.81 (1.60, 5.67)	38,793 (16,123, 57,863)	83.94 (34.95, 125.16)	3,020 (1,248, 4,598)	3.39 (1.40, 5.17)	65,167 (26,905, 99,425)	75.90 (31.31, 115.88)	−0.35 (−0.57, −0.13)	−0.31 (−0.54, −0.07)
Southern Sub-Saharan Africa	347 (146, 543)	1.38 (0.58, 2.15)	9,460 (3,979, 14,869)	32.51 (13.66, 50.92)	969 (409, 1,476)	1.81 (0.76, 2.74)	26,263 (11,059, 39,903)	42.77 (18.08, 65.07)	0.92 (0.53, 1.32)	0.90 (0.61, 1.19)
Tropical Latin America	1,427 (590, 2,143)	1.69 (0.70, 2.55)	38,033 (15,692, 56,901)	39.42 (16.28, 59.13)	5,029 (2,144, 7,573)	1.98 (0.84, 2.98)	126,986 (54,321, 191,291)	48.70 (20.82, 73.35)	0.54 (0.31, 0.78)	0.73 (0.58, 0.88)
Western Europe	24,785 (10,511, 36,960)	4.18 (1.77, 6.23)	510,578 (215,360, 765,413)	90.32 (38.19, 135.28)	28,985 (11,875, 43,653)	2.79 (1.16, 4.19)	538,523 (222,514, 804,551)	60.30 (24.79, 89.42)	−1.30 (−1.41, −1.18)	−1.32 (−1.45, −1.19)
Western Sub-Saharan Africa	753 (307, 1,152)	0.95 (0.39, 1.45)	19,755 (8,019, 30,293)	21.74 (8.89, 33.34)	1833 (788, 2,757)	1.07 (0.46, 1.60)	48,528 (20,549, 73,776)	23.49 (10.12, 35.34)	0.40 (0.34, 0.45)	0.26 (0.17, 0.34)

**Figure 1:**
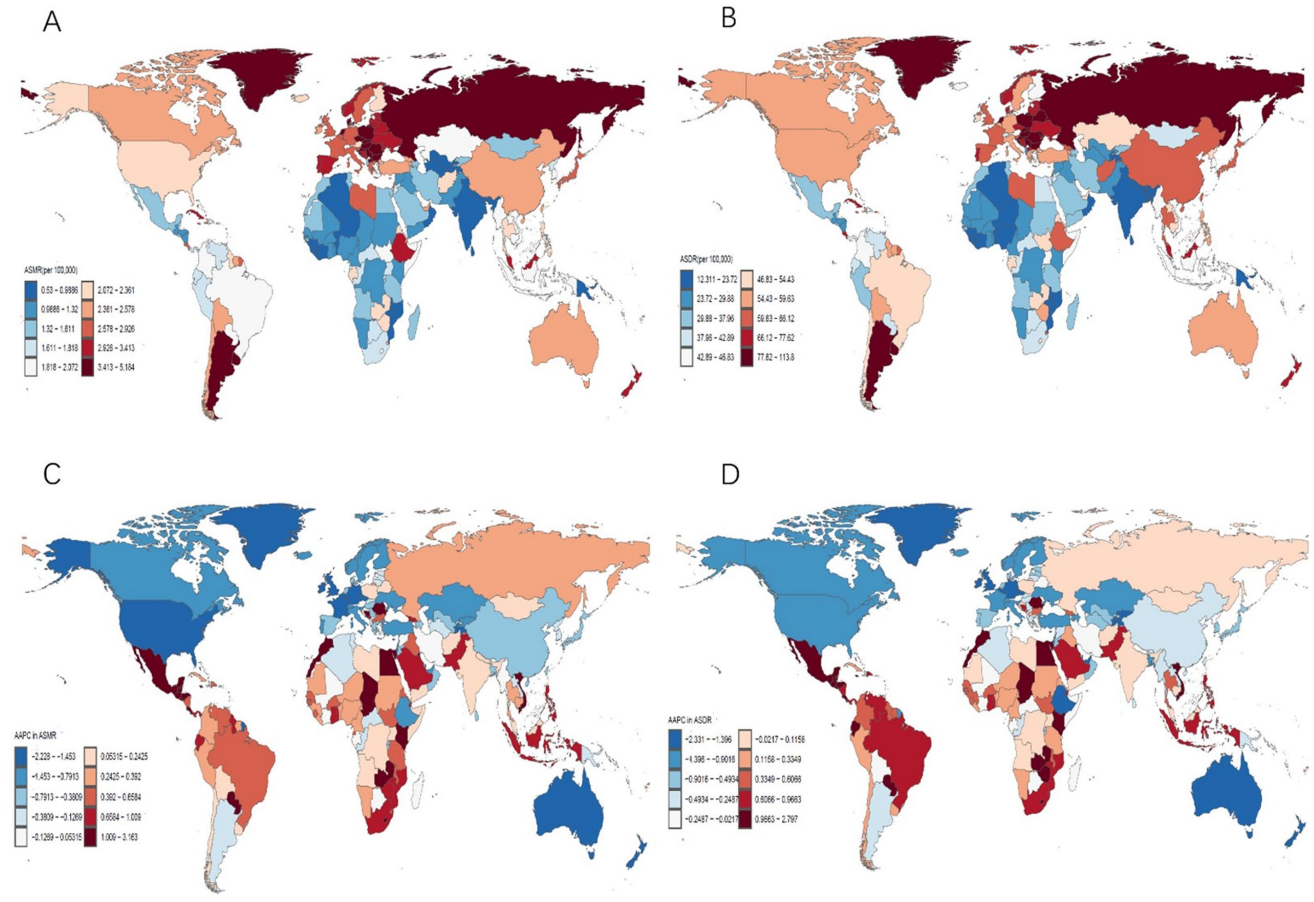
ASMR **(A)** and ASDR **(B)** for CRC attributable to low whole grains intake in 204 countries in 2021. The AAPC of the ASMR **(C)** and ASDR **(D)** for CRC attributable to low whole grains intake from 1990 to 2021; ASMR, age-standardized mortality rate; ASDR, age-standardized DALYs rate; AAPC, average annual percentage change; CRC, colorectal cancer.

In 2021, China, the United States, and Japan ranked the top three for CRC deaths attributable to low whole grains intake, while China, the US, and India ranked the top three for CRC DALYs. In 2021, Uruguay had the highest ASMR at 5.18 (95% UI: 2.09 to 7.73) per 100,000, and Hungary had the highest ASDR at 113.76 (95% UI: 47.61 to 173.37) per 100,000. However, Cabo Verde experienced the greatest increases from 1990 to 2021, with AAPCs of 3.16 (95% CI: 2.37 to 3.96) for ASMR and 2.80 (95% CI: 2.09 to 3.51) for ASDR (Figure 1).

### 3.2 Joinpoint regression analysis

**Figure 2:**
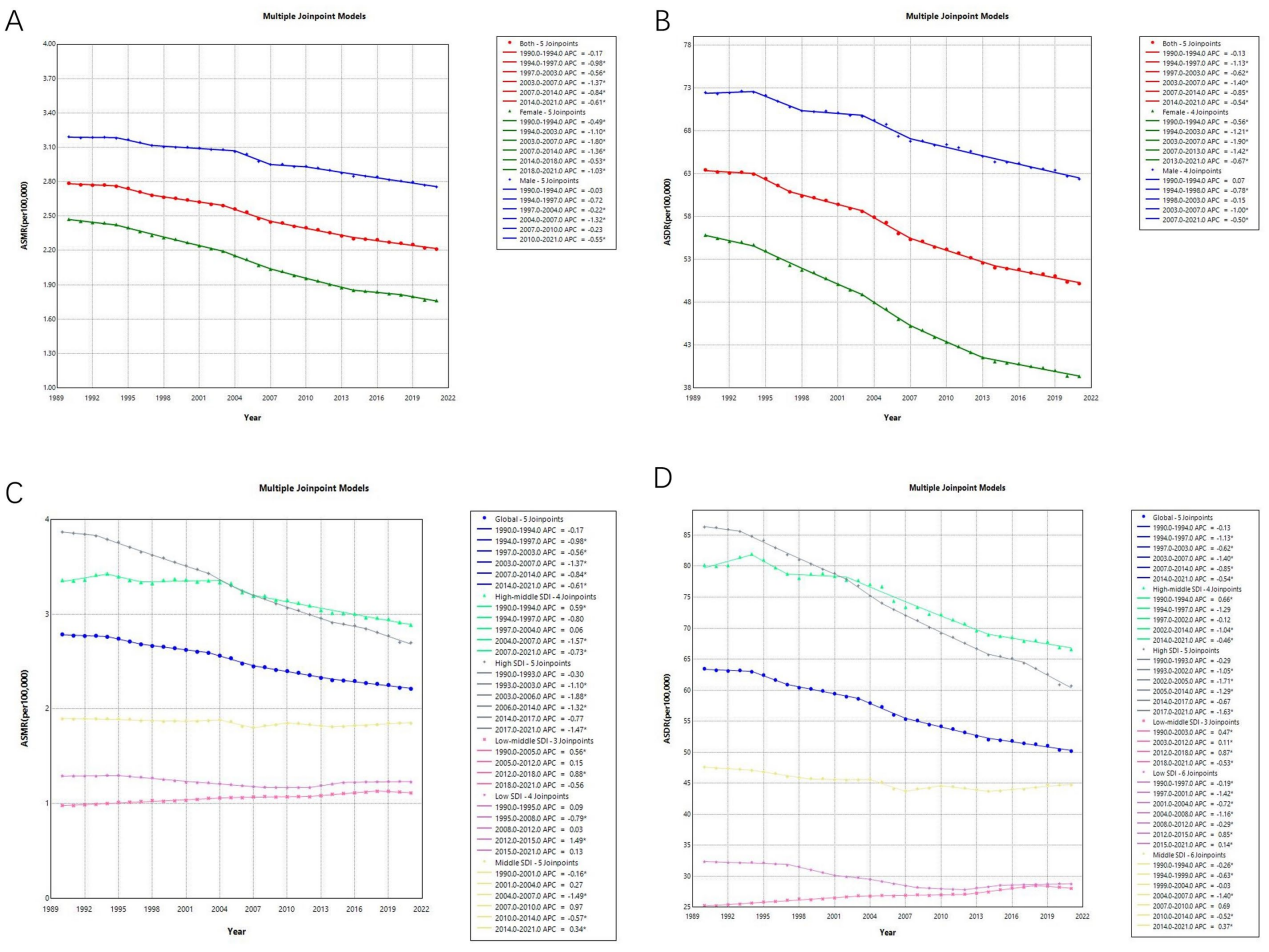
Temporal trends of CRC ASMR and ASDR attributed to low whole grains intake by joinpoint regression, 1990–2021. * means *p*-values < 0.05 and significant results. Data was provided for global, male, and female ASMRs **(A)**, global, male, and female ASDRs **(B)**, global and different SDI region ASMRs **(C)** and global and different SDI region ASDRs **(D)**. CRC, colorectal cancer; ASMR, age-standardized mortality rates; ASDR, age-standardized DALYs rates.

Figure 2 illustrates the Joinpoint regression trends in ASMR and ASDR for CRC due to low whole grains intake among global males, females, and five SDI regions from 1990 to 2021. Globally, the ASMR for all patients, male patients, and female patients shows a steady decline, with a smaller decrease in male patients (AAPC: −0.47, 95% CI: −0.59 to −0.34, *p* < 0.001) and a more significant decrease in female patients (AAPC: −1.09, 95% CI: −1.19 to −0.99, *p* < 0.001). Regarding SDI regions, different SDI areas exhibit varying trends. In high SDI regions, the ASMR shows a significant declining trend (AAPC: −1.17, 95% CI: −1.31 to −1.04, *p* < 0.001). In middle SDI regions, the overall trend of ASMR remains stable (AAPC: −0.08, 95% CI: −0.24 to 0.09, *p* = 0.34), while in low-middle SDI regions, an increasing trend is observed (AAPC: 0.42, 95% CI: 0.33 to 0.51, *p* < 0.001) (Table 1 and Figures 2A,C).

Similar to the trend in ASMR, the ASDR for global patients, as well as male and female patients, shows a significant declining trend. Likewise, regarding SDI regions, the trend in ASDR changes remains similar to that of ASMR, with the most significant decline observed in high SDI regions (AAPC: −1.15, 95% CI: −1.28 to −1.01, *p* < 0.001), while low-middle SDI regions exhibit a notable increasing trend (AAPC: 0.35, 95% CI: 0.29 to 0.40, *p* < 0.001) (Table 1 and Figures 2B,D).

### 3.3 Age and sex CRC burden attributable to low whole grains intake

**Figure 3:**
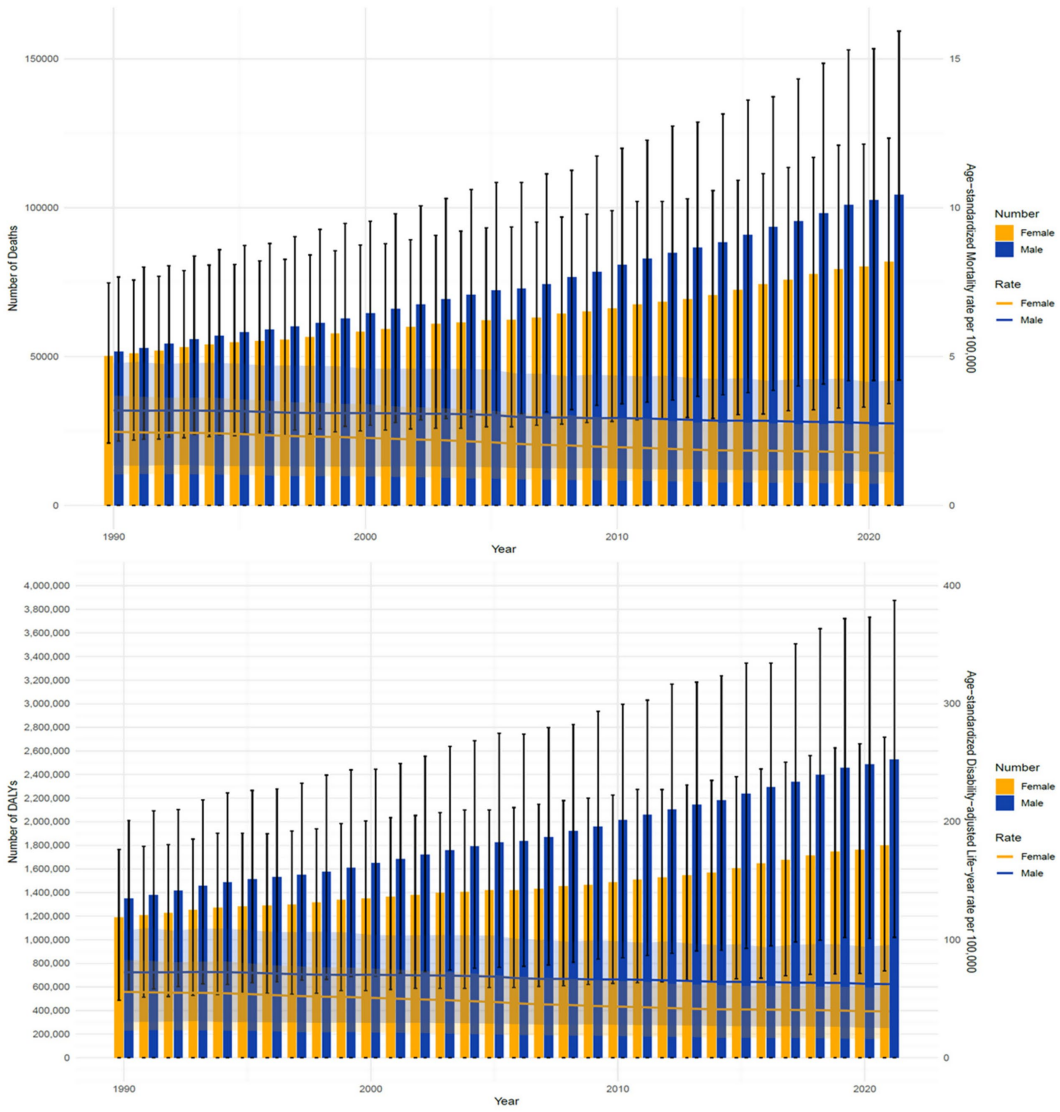
Comparison of full-age cases ASMR and ASDR of CRC attributable to low whole grains intake among male and female from 1990 to 2021. CRC, colorectal cancer.

From 1990 to 2021, the ASMR for CRC due to low whole grains intake showed a significant decline in both males and females, with males decreasing from 3.19 (95% UI: 1.35 to 4.78) per 100,000 to 2.75 (95% UI: 1.11 to 4.2) per 100,000, and females decreasing from 2.47 (95% UI: 1.04 to 3.69) per 100,000 to 1.76 (95% UI: 0.74 to 2.66) per 100,000. However, the number of deaths exhibited an increasing trend year by year. A similar pattern was observed in the ASDR. During this period, the ASDR for male CRC decreased from 72.49 (95% UI: 30.36 to 107.77) per 100,000 to 62.39 (95% UI: 25.20 to 95.64) per 100,000, while the ASDR for females decreased from 55.85 (95% UI: 22.91 to 82.83) per 100,000 to 39.36 (95% UI: 16.10 to 59.45) per 100,000. However, the DALYs exhibited an increasing trend year by year (Table 1 and Figure 3).

In 2021, the number of deaths from CRC due to low whole grains intake among global males and females showed a trend of initially increasing and then decreasing with age. In all age groups under 84, the number of deaths from CRC due to low whole grains intake in males consistently exceeded that in females, highlighting the higher exposure risk for males under 84. In the age group over 84, females deaths surpassed those of male. Males had the highest number of deaths in the 70–74 age group, while females had the highest number in the 80–84 age group, with males mortality rates consistently higher than those of females. Similarly, DALYs also exhibited a trend of initially increasing and then decreasing with age, highlighting the significant impact of CRC due to low grains diets on the lifespan of middle-aged and elderly populations. In all age groups under 84, males consistently had higher DALYs, further emphasizing the heavy burden of CRC on males. However, there were no significant changes in those under 45. The DALYs for both males and females were most severe in the 65–69 age group.

From a gender perspective, female mortality rates gradually increased starting at age 25, with an acceleration after 40, becoming more pronounced after 60, and peaking beyond age 95. In contrast, male mortality rates began to rise around age 30 and surged significantly after 45, indicating an earlier onset of increased risk compared to females.

**Figure 4:**
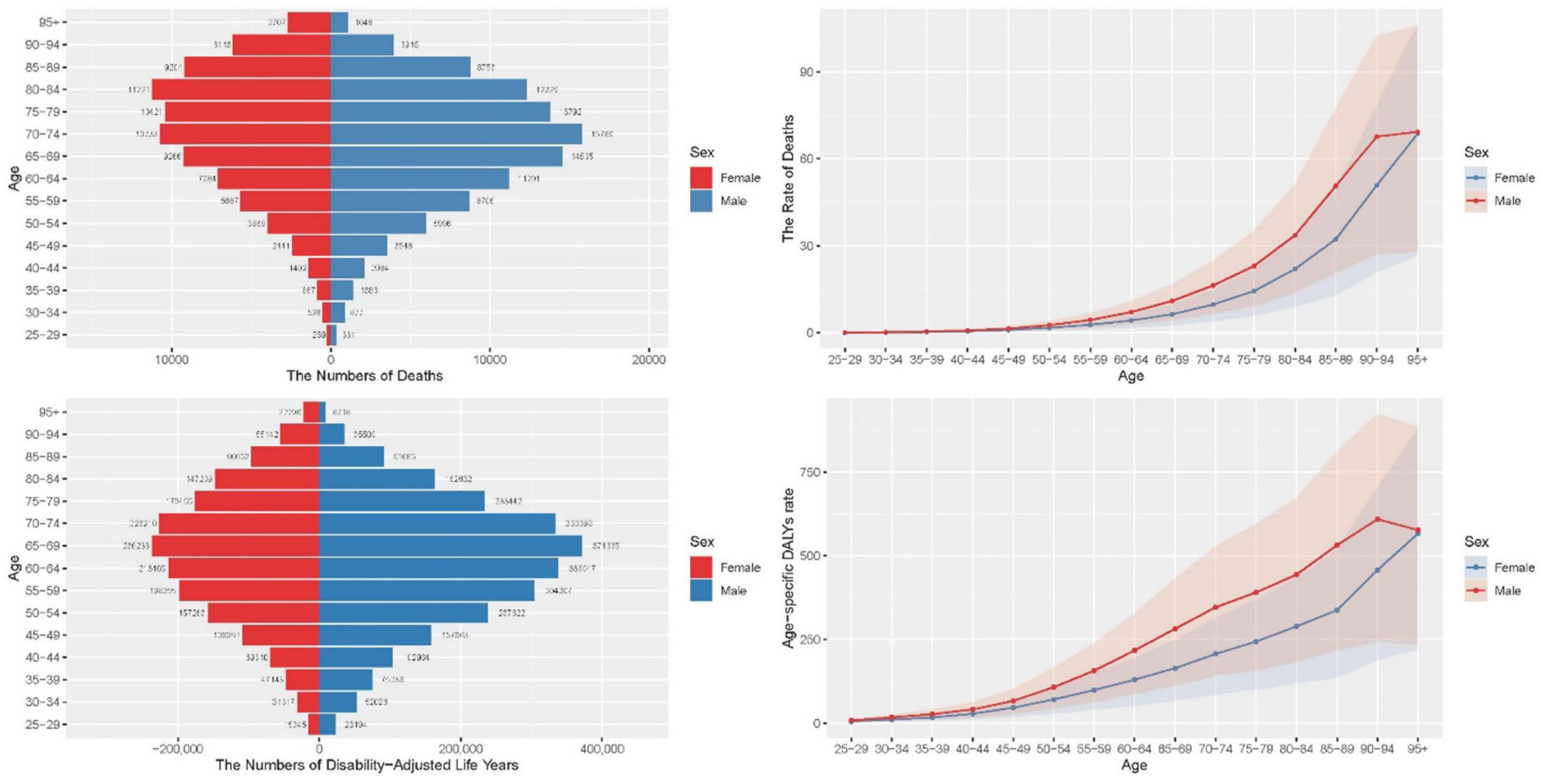
Comparison of the burden of CRC attributable to low whole grains intake in different age groups and genders in 2021. CRC, colorectal cancer.

Similarly, for females, the rate of DALYs was close to zero at age 25, then began to rise slowly, accelerated after the age of 40, and peaked at the age of 95. The trend for males is similar, but the rate of DALYs began to increase earlier and more rapidly, remaining at a higher level in most age groups, indicating a faster accumulation of health loss due to CRC in males. Compared to males, females had lower mortality and DALYs rates across all age groups, indicating a higher burden of CRC in males. However, with increasing age, the gender gap gradually narrows, especially after the age of 90, where the mortality rate and DALYs for females increase at a higher rate than for males. This may be due to the longer life expectancy of females and specific health risks in later life. The results are illustrated in Figure 4.

### 3.4 CRC burden attributable to low whole grains intake association with the SDI

**Figure 5:**
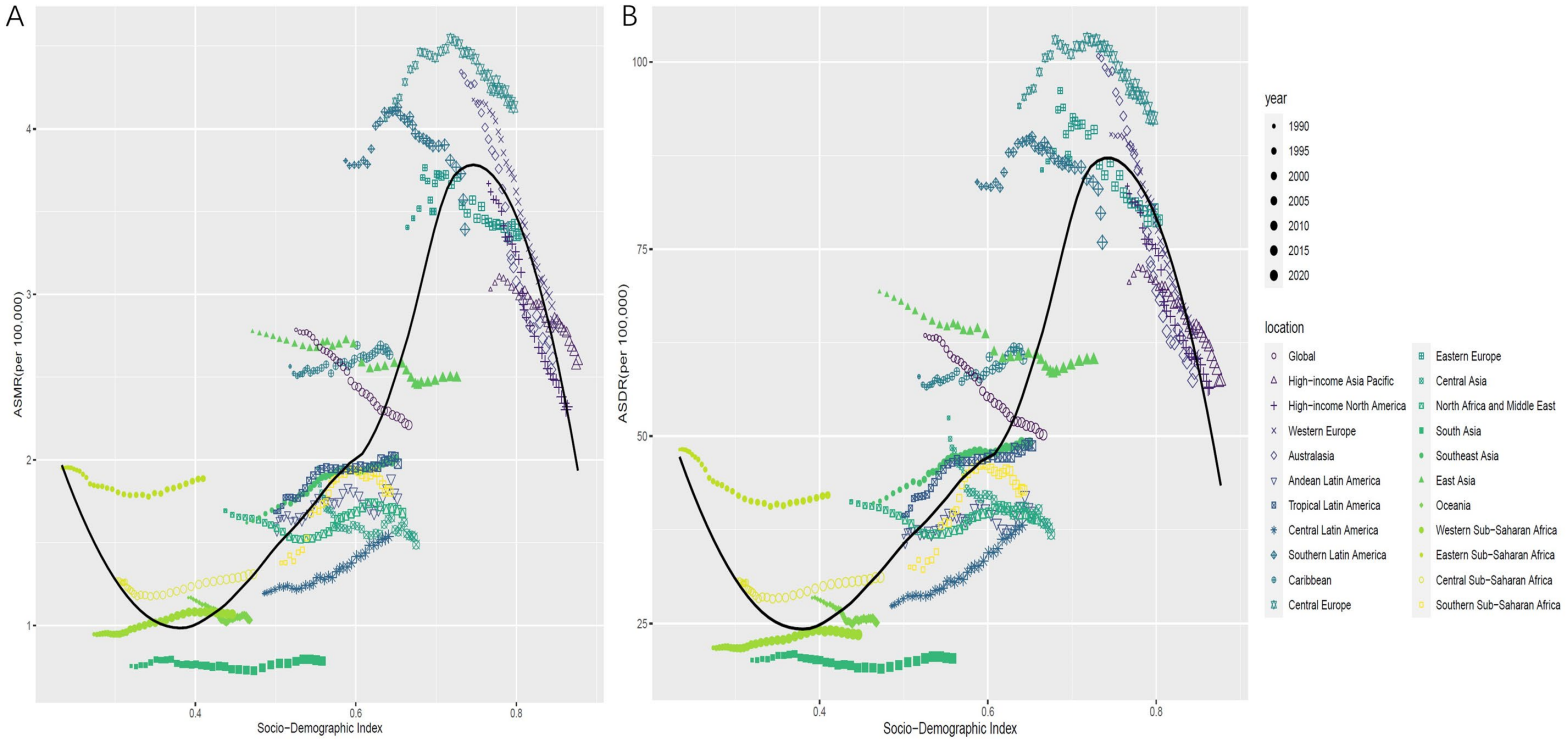
The correlation between ASMR for CRC attributable to low whole grains intake in 21 GBD regions from 1990 to 2021 and SDI **(A)**. The correlation between ASDR for CRC attributable to low whole grains intake in 21 GBD regions from 1990 to 2021 and SDI **(B)**. ASMR, age-standardized mortality rate; ASDR, age-standardized DALYs rate; SDI, sociodemographic index; CRC, colorectal cancer.

**Figure 6:**
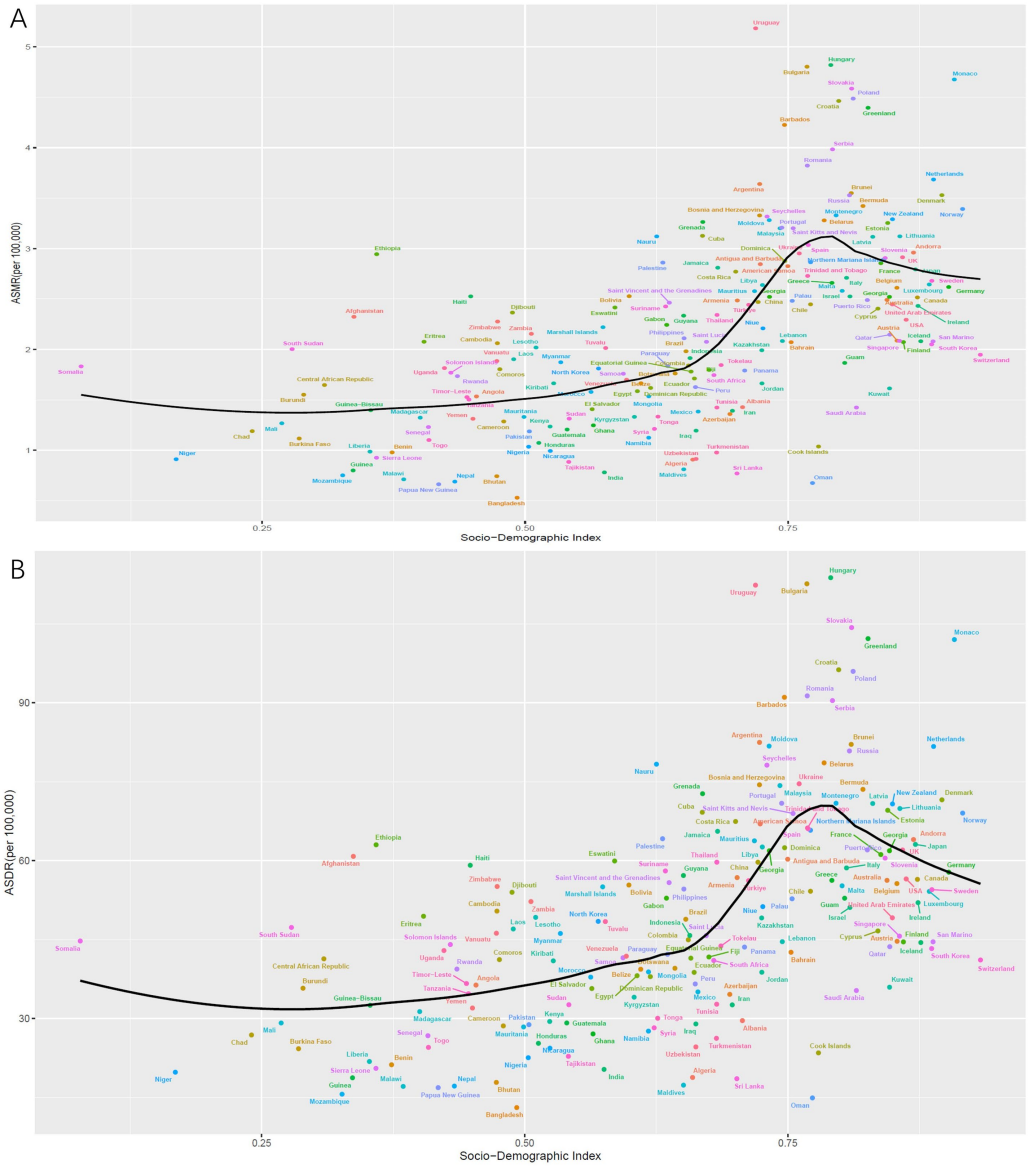
The correlation between ASMR for CRC attributable to low whole grains intake in 204 countries in 2021 and SDI **(A)**. The correlation between ASDR for CRC attributable to low whole grains intake in 204 countries in 2021 and SDI **(B)**. ASMR, age-standardized mortality rate; ASDR, age-standardized DALYs rate; SDI, sociodemographic index; CRC, colorectal cancer.

Overall, there is a nonlinear “S” shaped association between the ASMR for CRC due to low whole grains intake and the SDI. The ASMR gradually decreases when SDI is less than 0.38, rapidly increases between 0.38 and 0.74, peaks at an SDI of 0.74, and then rapidly decreases when SDI is greater than 0.74. Among different regions, the highest ASMR associated with low whole grains intake was observed in Central Europe, followed by Southern Latin America and Eastern Europe, while the lowest ASMR associated with low whole grains intake was found in South Asia and Oceania (Figure 5A). A similar association exists between the ASDR and SDI (Figure 5B). In 2021, among 204 countries and regions worldwide, the relationship between the ASMR and ASDR for CRC attributable to low whole grains intake and SDI initially decreased, then slowly increased with rising SDI. It began to increase rapidly when SDI exceeded 0.67, peaked at an SDI of 0.79, and then started to decline (Figures 6A,B).

**Figure 7:**
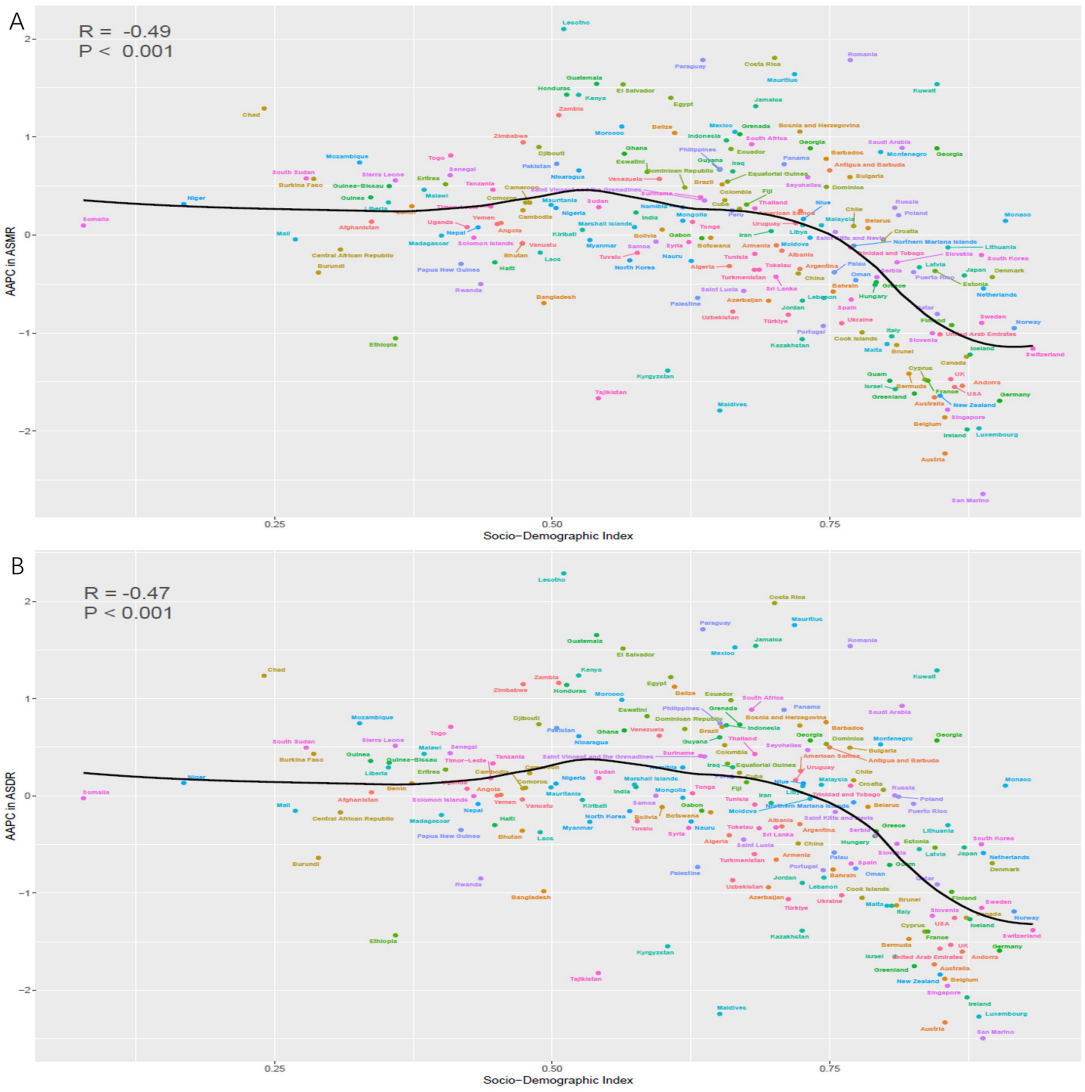
The correlation between the AAPC of ASMR for CRC attributable to low whole grains intake and SDI in 2021 **(A)**. The correlation between the AAPC of ASDR for CRC attributable to low whole grains intake and SDI in 2021 **(B)**. ASMR, age-standardized mortality rate; ASDR, age-standardized DALYs rate; SDI, sociodemographic index; AAPC, average annual percentage change; CRC, colorectal cancer.

From 1990 to 2021, a significant negative correlation was observed between the AAPC of ASMR and SDI (*R* = −0.49, *p* < 0.001) (Figure 7A). Similarly, there was an inverse relationship between the AAPC of ASDR and the SDI of various countries (*R* = −0.47, *p* < 0.001) (Figure 7B).

### 3.5 Frontier analysis of CRC burden attributable to low whole grains intake

**Figure 8:**
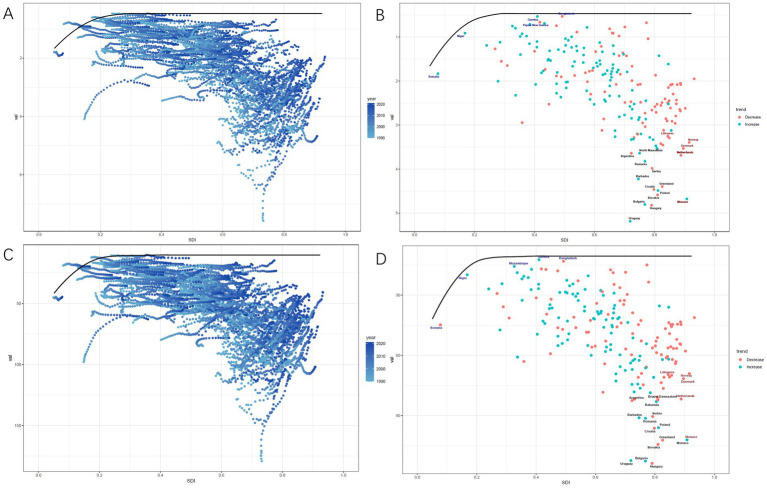
Frontier analysis results of CRC attributable to low whole grains intake ASMR and ASDR. Frontier analysis of ASMR from 1990 to 2021 **(A,B)**. Frontier analysis of ASDR from 1990 to 2021 **(C,D)**. Black lines represent the lower limits of ASRs achievable at different SDI levels, with points representing different countries and regions. The 15 countries and regions with the largest effective differences globally are labeled in black font, the 5 countries and regions with the smallest effective differences among low SDI countries are labeled in blue font, and the 5 countries and regions with the largest effective differences among high SDI countries are labeled in red font. In **(A,C)**, the blue dots represent the ASRs of CRC from 1990 to 2021, with darker shades indicating later years. In **(B,D)**, the dots represent changes in CRC ASR from 1990 to 2021. Blue dots indicate countries and territories where the ASR increased from 1990 to 2021, while red dots indicate countries and territories where the ASR decreased. SDI, Socio-demographic index; ASMR, age-standardized mortality rate; ASDR, age-standardized DALYs rate; CRC, colorectal cancer; ASR, age-standardized rate.

To better understand the potential improvements in the burden of CRC due to low whole grains intake, we conducted a frontier analysis based on ASMR, ASDR, and the SDI levels of regions and countries from 1990 to 2021. The frontier line shows the lowest achievable ASRs for each country or region at corresponding SDI levels, becoming stable when SDI surpasses 0.27. The gap between individual points and this black line reflects the potential for reducing future disease burden, with larger gaps indicating greater opportunities. In the four diagrams, these points form an inverted triangle, with lower values on the left and higher values on the right. As SDI increases, the variation in point distribution widens, indicating that countries with higher SDI have greater potential to lower CRC ASMR and ASDR linked to low whole grains intake. The trends in changes of ASMR and ASDR from 1990 to 2021 are shown in Figures 8A,C. For CRC ASMR due to low whole grains intake, Bangladesh, Gambia, and Papua New Guinea are the top three countries with the highest SDI and the closest effective distances, demonstrating the best ASMR control in these countries. Interestingly, we found that the 10 countries with the largest effective distances for ASMR have SDI levels above 0.70. These countries are Uruguay, Hungary, Bulgaria, Monaco, Slovakia, Poland, Croatia, Greenland, Barbados, and Serbia (Figure 8B). The frontier for ASDR also shows a similar trend (Figure 8D).

### 3.6 APC analysis of CRC burden attributable to low whole grains intake

**Figure 9:**
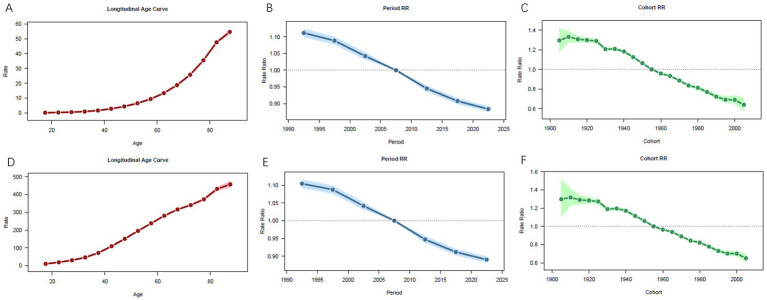
Age-period-cohort model analysis of CRC attributable to low whole grains intake. **(A–C)** Age period and cohort effect of deaths rate. **(D–F)** Age period and cohort effect of DALYs rate; CRC, colorectal cancer.

Under the assumption of controlling for period and cohort effects, from 1990 to 2021, the globally CRC deaths rates and DALYs rates due to low whole grains intake increased with age, reaching a peak in the age group over 95 years (Figures 9A,D). After controlling for age and cohort effects, from 1990 to 2021, the period RR values for CRC mortality and DALYs rates attributable to low whole grains intake globally showed a decreasing trend over time, using the 2007–2011 period as the reference group (RR = 1). The highest mortality RR was observed in 1992–1996 at 1.11 (95% CI 1.10–1.12) (Figure 9B); similarly, the DALYs rate RR peaked at 1.10 (95% CI 1.09–1.12) during 1992–1996 (Figure 9E). After controlling for age and period effects, the cohort RR values for CRC mortality and DALYs rates linked to low whole grains intake globally from 1990 to 2021 initially increased, then fluctuated with a declining trend over time. Using the 1955–1959 birth cohort as the reference group (RR = 1), the highest mortality RR was observed in the 1910–1914 cohort at 1.33 (95% CI 1.28–1.39) (Figure 9C); similarly, the DALYs rate RR peaked at 1.32 (95% CI 1.23–1.41) during this period (Figure 9F).

### 3.7 Projections of ASRs of CRC attributable to low whole grains intake to 2046

**Figure 10:**
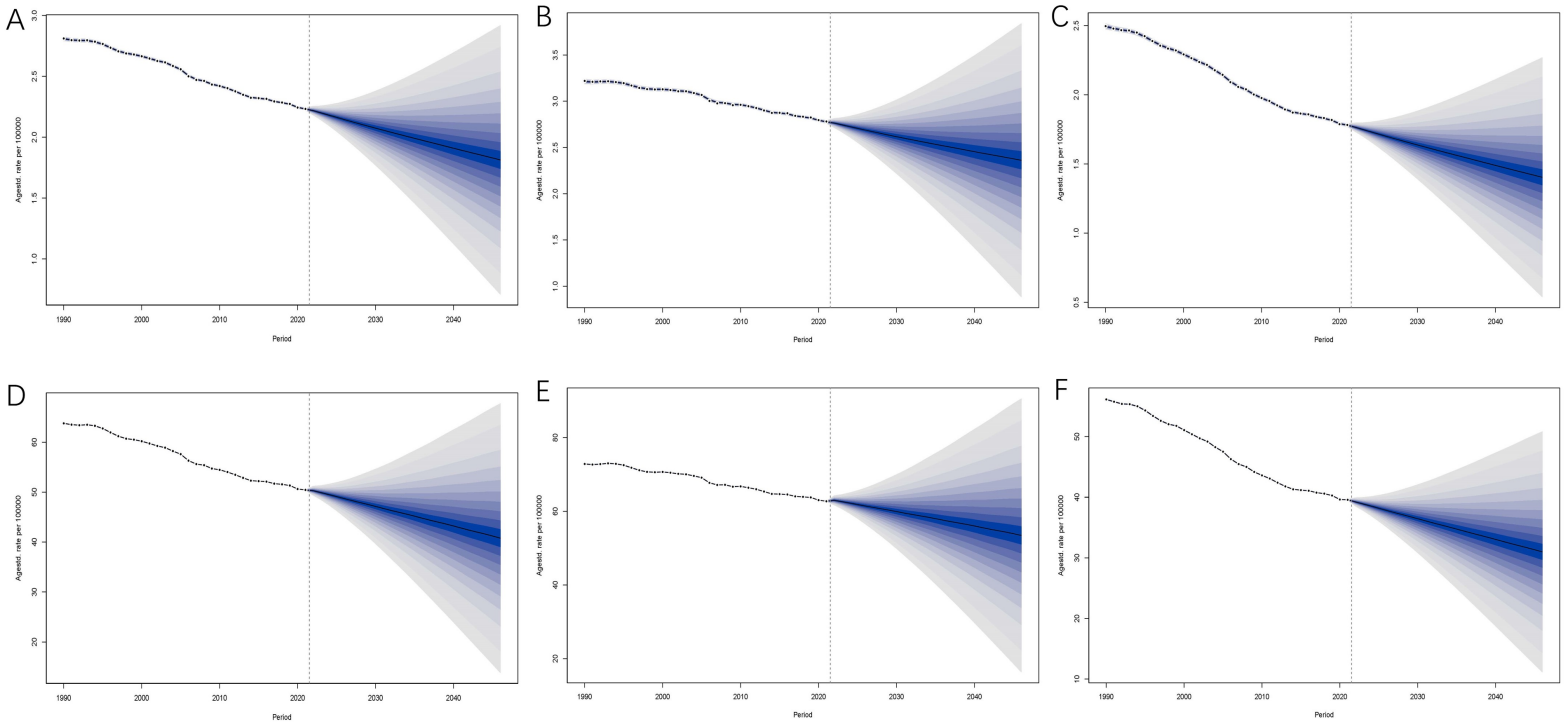
Global trends in ASRs of CRC attributable to low whole grains intake by gender from 1990 to 2046. **(A–C)** ASMRs for all sexes, males, and females. **(D–F)** ASDRs for all sexes, males, and females; ASRs, age-standardized rates; CRC, colorectal cancer; ASMRs, age-standardized mortality rates; ASDR, age-standardized DALYs rate.

Using the BAPC model, we predicted the trends of ASMR and ASDR for CRC due to low whole grains intake from 2022 to 2046 by gender. The predictions indicate a reduction in ASMR to varying degrees in both sexes, with ASDR expected to decrease in both male and female groups, showing similar trends. At the same time, the predictive analysis shows that both ASMR and ASDR decrease at a significantly faster rate in females compared to males (Figure 10).

## 4 Discussion

The CRC ranks third among newly diagnosed cancers worldwide, imposing a significant disease burden on the global population (4). Dietary factors play a crucial role in its etiology and progression; for instance, low whole grains intake, high-fat diets, and high consumption of processed meats all promote the development of CRC (32–34). Insufficient whole grains intake has been the leading cause of CRC-related deaths since 1990. However, studies examining the long-term trends of the burden of CRC attributable to low whole grains diets remain limited. This study systematically analyzed the global changes in the burden of CRC due to low whole grains intake from 1990 to 2021, examined its associations with the SDI, gender, age, and geographic regions, and conducted frontier and predictive analyses. The aim of this study is to elucidate the evolving trends in the impact of low whole grains intake on CRC burden, providing evidence to inform targeted nutritional interventions and public health strategies.

The study found that, despite significant increases in the absolute numbers of CRC deaths and DALYs attributable to low whole grains intake globally—rising by 82.94 and 70.30%, respectively—ASMR and ASDR showed decreasing trends. This apparent contradiction may be due to population growth and aging offsetting improvements in age-standardized rates. Notably, significant differences exist among SDI regions and genders: high-SDI regions experienced the most pronounced declines in ASMR and ASDR, while middle-to-low SDI regions showed increasing trends. Additionally, worldwide, the disease burden is higher in males than in females; however, females exhibit faster decreases in ASMR and ASDR, and among the elderly (>84 years), female deaths and DALYs surpass those of males. Geographically, the highest absolute burdens are observed in East Asia, Western Europe, and high-income North America, while countries such as Uruguay and Hungary have the highest age-standardized rates globally.

The findings of this study are consistent with previous epidemiological research linking diet and CRC, indicating a negative association between whole grains intake and CRC risk. Some studies have demonstrated a dose–response relationship, whereby each additional serving of whole grains is associated with approximately a 17.00% reduction in CRC risk (12, 35–37). Moreover, this study further reveals heterogeneity in temporal trends: high-SDI countries may have reduced disease burden through improved dietary patterns, increased screening, and advances in medical technology, while middle- and low-SDI countries may experience increasing burdens due to Westernized diets, increased processed food consumption, and insufficient public health resources. Unlike studies focusing solely on absolute case numbers, this study employed age-standardized rates to eliminate the effects of population structure, thereby providing a more accurate reflection of disease prevention and control effectiveness. Additionally, the faster decline in ASMR and ASDR among females compared to males may be related to increased health awareness, the protective effects of estrogen, and differences in gut microbiota; however, further validation is needed (38–41).

The study suggests that whole grains diets may inhibit CRC development through multiple mechanisms. First, whole grains are rich in insoluble fiber, which increases stool bulk, shortens intestinal transit time, and reduces contact between carcinogens (such as secondary bile acids) and the intestinal mucosa (42, 43). Second, components such as polyphenols, vitamin E, and selenium in whole grains can suppress chronic inflammation (such as NF-κB pathway) and oxidative stress, both of which are key drivers of CRC (44–47). Finally, higher whole grains intake is associated with lower obesity rates and insulin resistance, both of which are independent risk factors for CRC (47–50). In summary, low whole grains intake may promote CRC development by reducing dietary fiber intake, enhancing chronic inflammation and oxidative stress, altering the gut microbiota, and exacerbating metabolic conditions—these factors collectively contribute to carcinogenesis.

This study provides critical evidence to guide public health strategies aimed at mitigating the CRC burden attributable to low whole grains intake. The revealed nonlinear, “S”-shaped relationship between SDI and disease burden offers a predictive framework for policymakers. It indicates that low-middle and middle SDI countries, currently experiencing the steepest rise in burden, stand at a critical juncture where proactive intervention during this nutritional transition phase is essential to avert a future surge in CRC cases. Conversely, high-SDI countries must sustain and intensify their efforts to maintain the observed declining trends. To translate these findings into action, a multi-tiered strategy is proposed. Firstly, at the national policy level, governments should implement fiscal policies—such as subsidizing whole grain production or taxing highly refined foods—to enhance affordability and accessibility. Complementing this, mandatory front-of-pack labeling and clear national standards for “whole-grain” products are crucial to empower informed consumer choices and prevent misleading marketing. National dietary guidelines and public awareness campaigns must also be updated to explicitly promote daily consumption targets (e.g., 140–160 g/day) and highlight the specific role of whole grains in cancer prevention. Secondly, healthcare systems must deliver targeted interventions. Regions with the highest burden, namely Eastern Europe and South America, should integrate dietary risk assessment and counseling into routine primary care and CRC screening programs. Within these regions, men and the elderly, identified as the highest-risk demographics, should be prioritized for screening and educational initiatives, with physicians trained to provide specific, actionable dietary advice. Finally, global coordination is key. International bodies like the WHO should incorporate whole grains intake targets into global non-communicable disease (NCD) frameworks and assist lower-SDI countries in building capacity for robust dietary surveillance. Furthermore, facilitating knowledge transfer from high-SDI countries with successful CRC prevention experiences to those earlier in their development trajectory could accelerate progress. Implementing these evidence-based, targeted policies can reshape food environments, guide individual choices, and ultimately contribute to a sustained reduction in the global CRC burden.

Previous studies have explored the relationship between low whole grain diet and the burden of CRC (27). Building on this prior work, our research employed advanced analytical methods, including the AAPC, Joinpoint regression analysis, Frontier analysis, APC models, and BAPC prediction models to provide a more comprehensive and nuanced understanding of the trends in disease burden. Despite these methodological improvements, our study has certain limitations. For instance, the data relied primarily on publicly available statistics, which may be subject to biases and incompleteness. Additionally, the quality of dietary intake data and cancer registry data at the national level varies, which may introduce estimation bias. Third, the analysis did not fully account for interactions with other dietary factors (such as red meat consumption) and lifestyle behaviors (such as smoking and physical activity). Lastly, ecological analysis cannot establish a direct causal relationship between whole grains intake and CRC. Collectively, these factors may contribute to biases in the results.

Future research should focus on: (1) conducting multicenter cohort studies integrating genomics and metabolomics to elucidate molecular mechanisms of whole grains’ protective effects; (2) developing regional dietary intervention models to evaluate economic costs and health benefits; (3) exploring synergistic effects between whole grains and other dietary components (such as prebiotics) to optimize nutritional recommendations. In summary, this study offers valuable insights into global CRC prevention and control, emphasizing that improving whole grains intake should be a central component of multidimensional public health strategies, while also drawing attention to nutritional inequalities during socioeconomic transitions. It also provides health implications for certain regions and countries, offering a theoretical foundation for implementing targeted measures.

## 5 Conclusion

This study systematically analyzed the impact of low whole grains intake on the burden of CRC at global, regional, and national levels, revealing complex spatiotemporal dynamics in the association between CRC and low whole grains intake as populations change and dietary habits evolve. The results indicate that, despite an overall downward trend in ASMR and ASDR globally, some regions, particularly low- and middle-income countries, have experienced increases. These findings suggest that low whole grains intake remains a significant modifiable risk factor for CRC and holds important implications for future public health interventions.

These findings highlight the potential preventive value of improving whole grains intake levels. Nutritional intervention strategies should be tailored to local contexts. Policymakers should enhance dietary health promotion and encourage the consumption of whole grains to mitigate the future burden of CRC. It is recommended to promote whole grains food production through tax incentives, require mandatory labeling of the proportion of whole grains on food packaging, and include whole grains intake as part of the national chronic disease surveillance indicators. Additionally, regional disparities suggest that differentiated public health measures are necessary for countries at various stages of development.

Future research should focus on evaluating the effectiveness of intervention strategies, exploring the mechanisms linking whole grains intake to CRC pathogenesis, and utilizing micro-level data to uncover individual differences. Through multidisciplinary collaborations, efforts can be made to optimize prevention strategies, thereby providing a solid scientific foundation for effective control of CRC and the promotion of overall population health.

## Data Availability

Publicly available datasets were analyzed in this study. This data can be found here: https://ghdx.healthdata.org/gbd-2021.

## Footnotes

1
https://vizhub.healthdata.org/gbd-results/
