# Correction: Trend and age-period-cohort analysis of colorectal cancer burden attributable to low whole grains intake: a global, regional, and national analysis with predictive modeling (1990–2046)

**DOI:** 10.3389/fnut.2026.1860794

**Published:** 2026-05-19

**Authors:** Kai Wang, Xiaodan Li, Zhiqiang Guo, Junsheng Chen, Yongsheng Li, Hongzhou Liu, Shuwei Guo

**Affiliations:** 1 Department of Colorectal Surgery, Heping Hospital Affiliated to Changzhi Medical College, Changzhi, Shanxi, China; 2 Department of Pediatric Health Care, Zhangzi County Maternal and Child Health Family Planning Service Center, Changzhi, Shanxi, China

**Keywords:** colorectal cancer (CRC), global burden of disease, low whole grains intake, death, disability-adjusted life years (DALYs), age-period-cohort (APC) model

The Figures were in the wrong order in the PDF version and the online version of this paper. The images of Figures 3, 4 were misplaced; the image intended for Figure 4 was shown as Figure 3 and the image intended for Figure 3 was shown as Figure 4, while the captions and descriptions are correct. The corrected Figures and their captions appear below.

**Figure 3:**
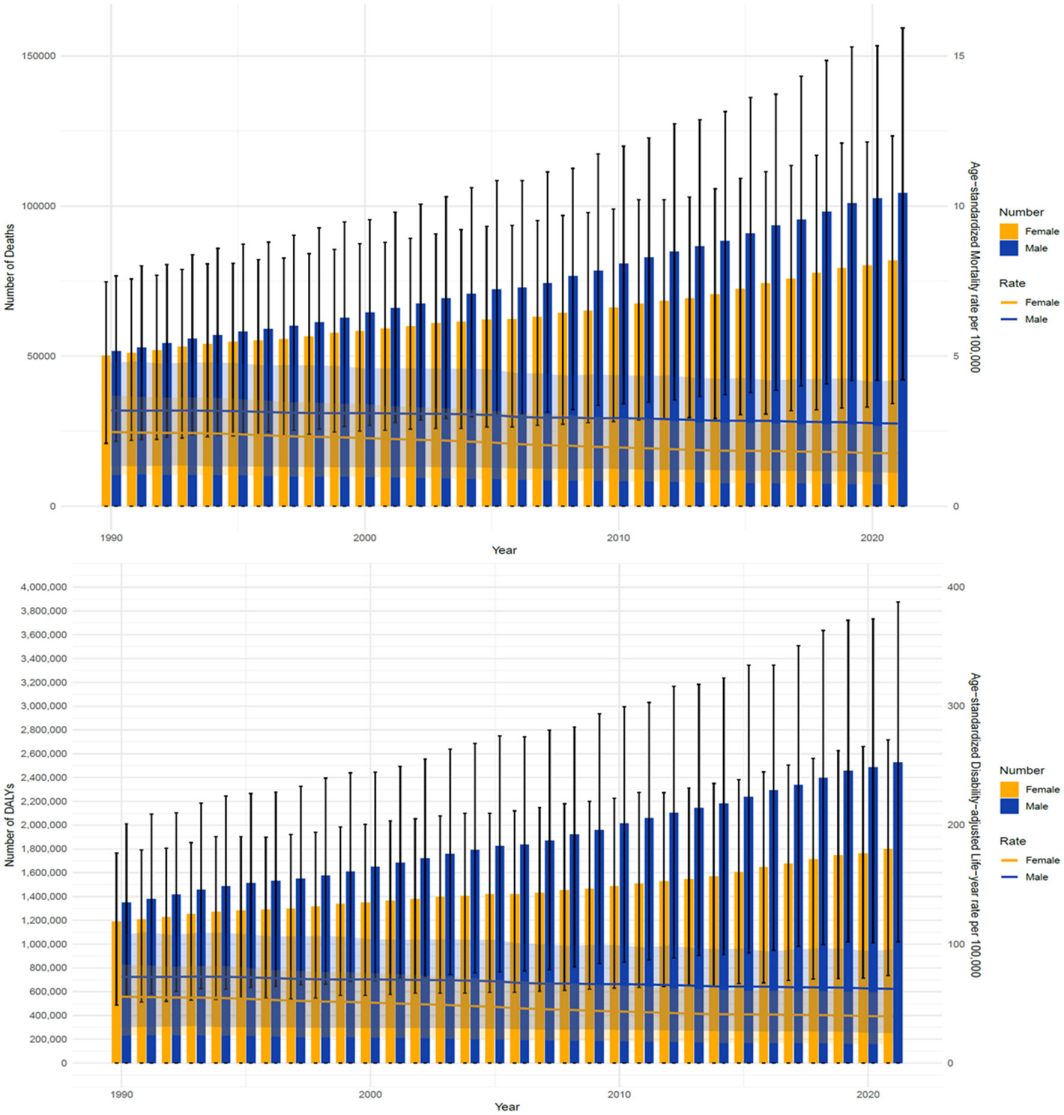
Comparison of full-age cases ASMR and ASDR of CRC attributable to low whole grains intake among male and female from 1990 to 2021. CRC, colorectal cancer.

**Figure 4:**
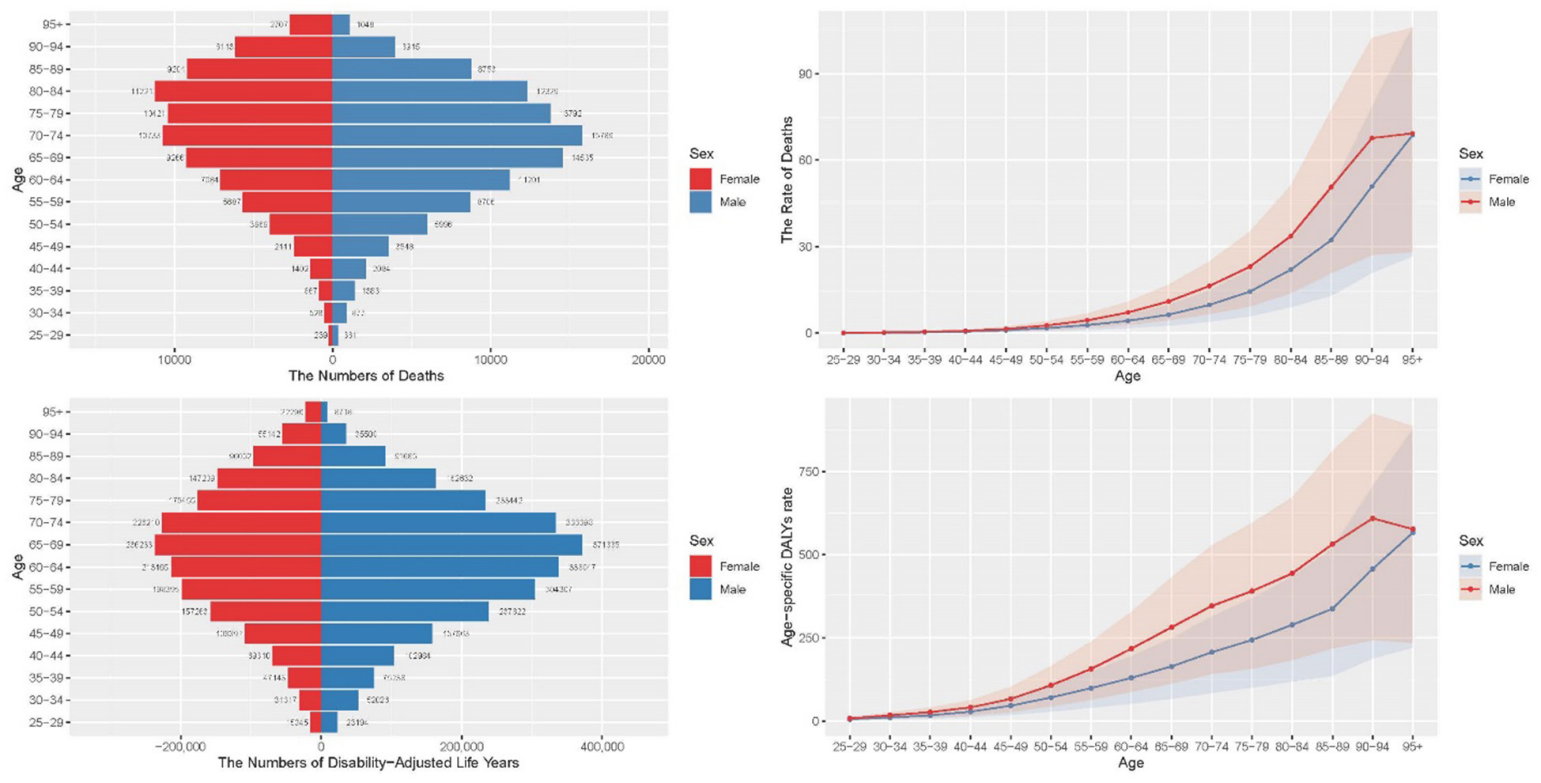
Comparison of the burden of CRC attributable to low whole grains intake in different age groups and genders in 2021. CRC, colorectal cancer.

The original version of this article has been updated.

